# mt tRFs, New Players in MELAS Disease

**DOI:** 10.3389/fphys.2022.800171

**Published:** 2022-02-22

**Authors:** Salvador Meseguer, Mari-Paz Rubio

**Affiliations:** Molecular and Cellular Immunology Laboratory, Centro de Investigación Príncipe Felipe (CIPF), Valencia, Spain

**Keywords:** sncRNAs, tRF and tiRNA, tRNA fragment, mitochondrial dysfunction, retrograde signaling

## Abstract

MELAS (mitochondrial encephalomyopathy, lactic acidosis, and stroke-like episodes) is an OXPHOS disease mostly caused by the m.3243A>G mutation in the mitochondrial tRNA^Leu(UUR)^ gene. Recently, we have shown that the mutation significantly changes the expression pattern of several mitochondrial tRNA-derived small RNAs (mt tsRNAs or mt tRFs) in a cybrid model of MELAS and in fibroblasts from MELAS patients versus control cells. Among them are those derived from mt tRNA LeuUUR containing or not the m.3243A>G mutation (mt 5′-tRF LeuUUR-m.3243A>G and mt 5′-tRF LeuUUR), whose expression levels are, respectively, increased and decreased in both MELAS cybrids and fibroblasts. Here, we asked whether mt 5′-tRF LeuUUR and mt 5′-tRF LeuUUR-m.3243A>G are biologically relevant and whether these mt tRFs are detected in diverse patient samples. Treatment with a mimic oligonucleotide of mt tRNA LeuUUR fragment (mt 5′-tRF LeuUUR) showed a therapeutic potential since it partially restored mitochondrial respiration in MELAS cybrids. Moreover, these mt tRFs could be detected in biofluids like urine and blood. We also investigated the participation of miRNA pathway components Dicer and Ago2 in the mt tRFs biogenesis process. We found that Dicer and Ago2 localize in the mitochondria of MELAS cybrids and that immunoprecipitation of these proteins in cytoplasm and mitochondria fractions revealed an increased mt tRF/mt tRNA ratio in MELAS condition compared to WT. These preliminary results suggest an involvement of Dicer and Ago2 in the mechanism of mt tRF biogenesis and action.

## Introduction

Mitochondria activity is genetically controlled by both mitochondrial and nuclear genomes. Mutations in those DNAs can lead to diseases owing to OXPHOS deficiency, which are accompanied with extremely variable clinical manifestations (Rotig, 2011; DiMauro et al., 2013; Boczonadi and Horvath, 2014). Mitochondria–nucleus communication influences mitochondrial disease expression and is considered an important contributor to such extreme variability (Reinecke et al., 2009; Varabyova et al., 2013; Dogan et al., 2014; Picard et al., 2014; Cagin and Enriquez, 2015; Chen et al., 2015). Small non-coding RNAs (sncRNAs) are recently emerging as players in mitonuclear cross-talk (Vendramin et al., 2017; Meseguer, 2021). Several studies have addressed the engagement of microRNAs (miRNAs) in the cell response to mitochondrial dysfunction (Meseguer et al., 2015, 2017, 2018; Wang et al., 2017); however, they seem not to be the unique type of sncRNAs that participate in the mitochondria–nucleus communication (Vendramin et al., 2017; Meseguer et al., 2019; Meseguer, 2021). tRNA-derived small fragments (tRFs or tsRNAs; ~16–35 nucleotides (nts) in length) are derived from nuclear- and mitochondrial-encoded mature and precursor tRNA sequences (Ro et al., 2013; Telonis et al., 2015; Olvedy et al., 2016; Pliatsika et al., 2016, 2018) and are generated constitutively or/and under certain conditions including cellular stress (Thompson et al., 2008; Thompson and Parker, 2009; Haussecker et al., 2010; Pederson, 2010; Garcia-Silva et al., 2012; Saikia et al., 2012; Gebetsberger and Polacek, 2013; Wang et al., 2013; Honda et al., 2015; Telonis et al., 2015; Megel et al., 2019). tRFs are arranged in major structural categories based on their sequence alignment respect to (a) the parental mature tRNA sequence: tRF-5s (also called 5′-tRFs), i-tRFs, tRF-2s, tRF-3s (or 3′-tRFs), 5′-tRNA halves (also known as 5′-tRHs or 5′-tiRNAs), and 3′-tRNA halves (3′-tRHs or 3′-tiRNAs) or (b) to the precursor tRNA in the 5′ leader (5′U-tRFs) or 3′ trailer (tRF-1s or 3′U-tRFs) sequences (Pliatsika et al., 2016; Loher et al., 2017; Xie et al., 2020). At present, there is not a full knowledge of the tRF biogenesis pathways operating in the cell, but some of the RNases reported for cytosolic tRFs are Angiogenin, RNase T2, Dicer, and RNaseZ/ELAC2 (Cole et al., 2009; Haussecker et al., 2010; Ivanov et al., 2011; Diebel et al., 2016; Megel et al., 2019; Su et al., 2019). Moreover, the function of tRFs is not yet clear. Since they can interact with various Argonaute (AGO) proteins and form biologically active complexes (Burroughs et al., 2011; Wang et al., 2013; Kuscu et al., 2018), tRFs could act as negative post-transcriptional regulators of specific mRNAs, as miRNAs do (Yeung et al., 2009; Kuscu et al., 2018).

Based on recent studies, mitochondrial tRFs differ from nuclear tRFs in terms of sequence and size (Telonis et al., 2015; Loher et al., 2018). New sensitive and specific methods like MINTmap take into consideration the existence of plenty of sequences in the nuclear genome matching mitochondrially encoded tRNAs (“mitochondrial tRNA-looklikes”) among other particularities of the tRNA sequences during the mapping of the RNA-seq data (Loher et al., 2017). Although there are several *in silico* studies, so far, there are few works providing experimental evidence about the mitochondrial origin and function of tRFs (Loher et al., 2017, 2018; Magee et al., 2018; Telonis and Rigoutsos, 2018; Meseguer et al., 2019; Londin et al., 2020).

In a previous study, we used a cybrid model of MELAS (mitochondrial encephalomyopathy, lactic acidosis, and stroke-like episodes) to demonstrate that the MELAS m.3243A>G mutation in the mitochondrial tRNALeu(UUR) gene, which is associated with OXPHOS dysfunction, significantly changes the production of specific mt tRFs in comparison to controls (Meseguer et al., 2019). The analysis of small RNA-seq data by MINTmap provided a list of differentially expressed mt tRFs in the comparison MELAS cybrids versus controls, some of which had unequivocal mitochondrial origin in basis of their sequence alignment on mtDNA, while that of others was considered uncertain because of their additional matching to several regions in the nuclear genome. Among the last group of ambiguous mt tRFs, there were two derived from the 5′-end of mt tRNA LeuUUR, containing or not the m.3243A>G mutation (mt 5′-tRF LeuUUR-m.3243A>G and mt 5′-tRF LeuUUR), whose expression levels were found, respectively, increased and decreased in MELAS cybrids and in fibroblasts from MELAS patients. Two other ambiguous mt tRFs studied were derived from the 3′-end of the mt tRNA ValUAC (mt 3′-tRF ValUAC) and from the internal region of mt tRNA GluUUC (mt i-tRF GluUUC). They followed the same expression pattern as mt 5′-tRF LeuUUR-m.3243A>G. Despite their ambiguous origin, they were verified as true mitochondrial products since (i) they were mainly detected in mitochondria after performing subfraction experiments which included an RNase treatment to eliminate any RNA anchored to the mitochondrial outer membrane and (ii) they had null expression in cells depleted of mtDNA but with the same background as MELAS cells (143B Rho0 cells).

Moreover, we reported that the levels of these selected mt tRFs were diminished after knocking down of Dicer and Argonaute 2, key components in the miRNA pathway. Following the hypothesis that some mt tRFs act as miRNAs, a tool for miRNA target prediction was used to identify potential mt tRFs targets. The functional analysis of these genes revealed connection between mt tRFs and processes involving the most common affected tissues in MELAS (neurological-, cardiac-, and muscular-related processes). However, we took a step beyond the *in silico* study and biological relevance of one of the mt tRFs (mt i-tRF GluUUC) was analyzed in the cybrid cells. In particular, we found that mt i-tRF GluUUC downregulates the expression of the mitochondrial pyruvate carrier 1 (MPC1) in MELAS cybrids, promoting the accumulation of extracellular lactate. Interestingly, levels of mt i-tRF GluUUC were dependent on mt tRNA modifying enzymes operating at wobble uridine position (U34) of the parental mt tRNA, mt tRNA GluUUC (GTPBP3, MTO1, and TRMU). MELAS cells exhibited increased levels of mt i-tRF GluUUC, at least in part due to downregulation of the mt tRNA modification enzymes by the post-transcriptional repressor activity of miR-9/9*, a stress-sensitive microRNA.

In the present work, we wanted to provide preliminary data on the biogenesis mechanism of mt tRFs, as well as new evidence of their biological relevance and their therapeutic/diagnostic potential. In particular, we determined whether Dicer and Ago2 are present in the mitochondria of the WT and MELAS cybrids. Furthermore, we wondered whether it is possible to detect mt tRNA and specific mt tRFs in the Dicer and Ago2 immunoprecipitates and, if so, if their levels change in the MELAS versus control comparison. We also studied whether restoring 5′-mt-tRFLeuUUR levels in MELAS cybrids could be of biological relevance by studying its possible effects on mitochondrial respiration. Finally, we tested the levels of a selected group of mt tRFs in a small cohort of MELAS samples of different types.

## Materials and Methods

### Materials

Oligonucleotides (Supplementary Table S1) were purchased from Sigma or Thermo Fisher.

### Cell Culture

Transmitochondrial cytoplasmic hybrids (cybrids) were prepared by fusing platelets derived from a patient carrying the m.3243A>G mutation with human osteosarcoma 143B cells lacking mtDNA (TK-; ρ0 cells) as previously described (Chomyn, 1996). They were cultured in high glucose Dulbecco’s modified Eagle medium (Gibco) containing 10% Fetal Bovine Serum (FBS), 1 mM sodium pyruvate, 100 U/ml penicillin, 100 μg/ml streptomycin, 2 mM glutamine, and 1 mM non-essential amino acids. They were kept at 37°C in a humidified atmosphere with 5% CO_2_.

### Ethics Statement

Muscle biopsies were provided by Telethon Network of Genetic Biobanks (Italy) and urine, plasma, and peripheral blood mononuclear cells (PBMCs) by Ciberer Biobank (Valencia, Spain). All samples were collected from patients with MELAS disease or from healthy subjects, and written informed consent was obtained from the participants. All procedures were approved by the Ethics Committee of Milano Area 2, Fondazione IRCCS Ca′ Granda Ospedale Maggiore Policlinico (Milan, Italy) and the Ethics Committee of Foundation for the Promotion of Health and Biomedical Research of Valencia Region, FISABIO (Valencia, Spain) and performed in accordance with the guidelines set forth by the Declaration of Helsinki.

### Fluorescence Microscopy

Cybrid cells were cultured on coverslips in 24-well plates. Twenty-four hour post-seeding, cells were rinsed with PBS, fixed with 4% paraformaldehyde–PBS for 20 min at room temperature (RT), washed with PBS, permeabilized with 0.3% Triton X-100 in PBS for 15 min and washed again with PBS. Then, unreacted paraformaldehyde was quenched in 100 mM NH_4_Cl, 150 mM glycine in PBS for 10 min, washed with PBS, and blocked with a solution containing 2% BSA and 0.05% Triton X-100 in PBS for 30 min at RT. Then, cells were incubated with 1:100-diluted anti-Ago2 (Sigma, SAB4200085-200UL) and anti-ATP5A1 (Thermo Fisher, 43-9800) or anti-Dicer (Abcam, ab14601) and anti-CLPP (Abcam, ab124822) antibodies in blocking solution overnight at 4°C. Upon washing with blocking solution, bound antibodies were detected by incubation, as appropriate, with 1:150-diluted Alexa Fluor 594-conjugated anti-rat (Invitrogen, A11007), Alexa Fluor 594-conjugated anti-mouse (A11020, Invitrogen), Alexa Fluor 488-conjugated anti-rabbit (Invitrogen, A11008), and Alexa Fluor 488-conjugated anti-mouse (A11001, Invitrogen) secondary antibodies in blocking solution for 1 h at 37°C. Slides were mounted in Prolong Gold antifade reagent with DAPI (Molecular Probes, 936,576), and images were obtained with Apotome-equipped Axio Observer Z1 microscope (Carl Zeiss AG).

### Subcellular Fractionation

3-5·10^7^ cybrid cells were harvested with trypsin–EDTA solution, washed with PBS, and resuspended in 1 ml ice-cold lysis buffer (0.6 M manitol, 1 mM EDTA, 10 mM pH 6.7 PIPES, 0.3% BSA) supplemented with 1 μg/ml PMSF and 1 μg/ml leupeptin. Cells were disrupted with a nitrogen cavitation pump (2.5 bar, 10 min), and cell lysate was passed through a glass Teflon Dounce homogenizer, ~20-times. Then, the cell lysate was subjected to centrifugation at 1,500 rpm for 5 min at 4°C. The supernatant (containing cytoplasm membrane and mitochondria) and pellet (containing nuclei and undisrupted cells) were separated, and the supernatant was further centrifuged at 20,000 *g* for 20 min. The clarified supernatant (cytoplasm and membrane fraction) and pellet (mitochondria) were separated. Supernatant was re-centrifuged at 20,000 *g* for 20 min to purify the cytoplasm fraction. Mitochondria pellet was kept in ice and treated with 100 μl of 4 mg/ml Digitonin in PBS for 10 min to remove the mitochondrial outer membrane and to obtain mitoplasts. Then, PBS was added up to 1.5 ml and centrifuged at 20,000 *g* for 20 min. Pellet was washed once with PBS. Cell extracts (nuclei + undisrupted cells, mitoplasts, and cytoplasm) were prepared in RIPA buffer (150 mM NaCl, 1% Nonidet P40, 0.5% sodium deoxycholate, 0.1% SDS, and 50 mM Tris–HCl pH 8.0), containing 0.1 mM leupeptin and 1 mM PMSF. Proteins (50 μg) from the various lysates were separated by SDS/PAGE (10% acrylamide) and transferred to PVDF membranes (GE Healthcare, Amersham Biosciences). For immunodetection, we used commercial antibodies: anti-Ago2 (Sigma, SAB4200085-200UL), anti-Dicer (Abcam, ab14601), anti-RNApol II (Santa Cruz, sc-899), anti-SDHA (Abcam, ab14715), and anti-vinculin (Santa Cruz, sc-73,614). Anti-rabbit (A6154), anti-rat (A5795), and anti-mouse (A4416) IgG-horseradish peroxidase-conjugated secondary antibodies were obtained from Sigma.

### Cell Culture Transfections

Cybrid cells were seeded at 1,500,000 cells/100 mm dish. After 24 h, they were transfected with antisense oligonucleotides targeting mt 5′-tRF LeuUUR-m.3243A>G (anti-mt 5′-tRF LeuUUR-m.3243A>G; custom mirVana miRNA inhibitor; Thermo Fisher), mimic molecules of mt 5′-tRF LeuUUR (pre-mt 5′-tRF LeuUUR; custom mirVana miRNA mimic; Thermo Fisher) or their respective negative Controls NC; mirVana^™^ miRNA Inhibitor, Negative Control #1 (NC-anti-mt tRF; 4464076; Thermo Fisher) and mirVana miRNA Mimic, Negative Control #1 (NC-pre-mt tRF; 4464058; Thermo Fisher) at a final concentration of 50 nM, using Lipofectamine 2000 reagent (Invitrogen) and Opti-MEM medium according to manufacturer’s instructions. The medium was replaced by fresh growth medium 6 h after transfection, and cells were collected 48 h after transfection.

### RNA Isolation and qRT-PCR

Total RNA from PBMCs, muscle samples, and MELAS cells transfected with mt 5′-tRF LeuUUR mimic or the control was isolated using TRIzol reagent (Invitrogen) and from plasma and urine samples using miRNeasy Serum/Plasma Kit (Qiagen) following the manufacturer’s protocol. For mt tRF quantification, 10 ng of total RNA were reverse-transcribed in 15 μl total reaction volume using the MultiScribe reverse transcriptase and custom miRNA specific stem-loop RT primers (Thermo Fisher). Then, 1.33 μl of the reverse transcription reaction was subjected to a custom TaqMan miRNA assay (Thermo Fisher), in a total reaction volume of 12 μl using specific primers and probes for the selected human mt tRFs and U6 snRNA, according to the manufacturer’s protocol. Expression values were calculated using the comparative CT method and U6 snRNA as an endogenous control. In case of plasma samples, the combination of miR-16, −191, and 484 was used as endogenous control. To quantify mitochondrial DNA-encoded transcripts, one-step qRT-PCRs were performed in an Applied Biosystems Step-One Real-Time PCR System. 5 μl of 1/10 diluted RNA samples were reverse-transcribed and amplified by qPCR in 12 μl of total volume reaction containing specific primers (Sigma; Supplementary Table S1), Power SYBR Green PCR Master Mix, MultiScribe Reverse Transcriptase, and RNase Inhibitor (all from Applied Biosystems), according to manufacturer’s instructions. Amplification efficiency values were very close to 100%. Expression values were calculated using the comparative CT method and 16S rRNA as an endogenous control.

### Dicer and Ago2 Immunoprecipitation

3-5·10^7^ cybrid cells were harvested with trypsin–EDTA solution, washed with PBS, and resuspended in growth medium with 1% formaldehyde. Cell suspensions were incubated at room temperature for 10 min on rocker. Unreacted formaldehyde was quenched by addition of Glycine to a final concentration of 141 mM and incubation at room temperature for 5 min on rocker. To pellet the cells, samples were centrifuged at 1,500 rpm for 5 min at 4°C. Medium was removed, and the pellet was washed with 2 ml cold PBS containing 0.1 mM leupeptin, 1 mM PMSF, and 20 U/ml RNase Inhibitor and resuspended in mitobuffer (0.6 M manitol, 1 mM EDTA, 10 mM PIPES pH 6.7, 0.3% BSA, 0.1 mM leupeptin, 1 mM PMSF, and 20 U/ml RNase Inhibitor). Cells were disrupted with a nitrogen cavitation pump (2.5 bar, 10 min), and cell lysates were passed through a glass Teflon Dounce homogenizer, ~20-times. Then, cell lysates were subjected to centrifugation at 1,500 rpm for 5 min at 4°C. The supernatant (containing cytoplasm membrane and mitochondria) and pellet (containing nuclei and undisrupted cells) were separated, and the supernatant was further centrifuged at 20,000 *g* for 20 min. The clarified supernatant (cytoplasm and membrane fraction) and pellet (mitochondria) were separated. Supernatant was re-centrifuged at 20,000 *g* for 20 min to purify the cytoplasm fraction. To eliminate the RNA anchored to mitochondria, mitochondria pellets were treated with 20 μg/ml RNaseA in 10 mM Tris pH 7.5 for 15 min at 37°C and washed with PBS containing 0.1 mM leupeptin, 1 mM PMSF, and 20 U/ml RNase Inhibitor. Mitochondria were resuspended in 1 ml mitochondria lysis buffer (25 mM HEPES-KOH pH 7.6, 10% Glycerol, 5 mM MgCl_2,_ 0.5 mM EDTA, 0.5% Tween 20, 0.15 M KCl, 0.1 mM leupeptin, 1 mM PMSF, and 20 U/ml RNase Inhibitor). After a 30 min incubation on ice, immunoprecipitation (IP) of crosslinked Protein/RNA was performed. For each immunoprecipitation (Ago2, Dicer, and IgG), 2 ml microfuge tubes containing 200 μl of mitochondrial lysates or cytoplasm fractions and 1.8 ml dilution buffer (16.7 mM Tris–HCl pH 8, 1.2 mM EDTA, 0.01% SDS, 1.1% Triton X-100, 165 mM NaCl, 0.1 mM leupeptin, 1 mM PMSF, and 20 U/ml RNase Inhibitor) were prepared. 20 μl Protein A Dynabeads (Invitrogen, 10002D) was added to each IP, and tubes were incubated for 2 h at 4°C with rotation. Dynabeads were pelleted using a magnet, and supernatants were transferred into new 2 ml microfuge tubes. Antibodies (10 μg) were added for each immunoprecipitation [IgG (Cell Signaling, 2729S), Ago2, and Dicer], and tubes were kept overnight at 4°C with rotation. Next day, 30 μl Protein A Dynabeads was added to each tube to collect the antibody/antigen/RNA complexes using the magnet. They were washed by resuspension in 1 ml of the following cold buffers in the order listed, low salt immune complex wash buffer (20 mM Tris–HCl pH 8, 150 mM NaCl, 1% Triton X-100, 2 mM EDTA, and 0.01% SDS), high salt immune complex wash buffer (20 mM Tris–HCl pH 8, 500 mM NaCl, 1% Triton X-100, 2 mM EDTA, and 0.01% SDS), LiCl immune complex wash buffer (10 mM Tris–HCl pH 8, 0.25 M LiCl, 1% NP40, 1 mM EDTA, and 1% deoxycholic acid), and TE buffer (10 mM Tris–HCl pH 8 and 1 mM EDTA). After the washes, antibody/antigen/RNA complexes were eluted by adding 250 μl elution buffer (100 mM NaHCO_3_ and 1% SDS) to each tube and tubes were incubated at RT mixing at 300 rpm for 15 min. Then, supernatants with antibody/antigen/RNA complexes were separated from beads with the magnet and transferred to 1.5 ml microfuge tubes. 750 μl TRIzol reagent was added to each tube, and RNA isolation was performed according to the manufacturer’s protocol. RNA pellets were resuspended in 30 μL RNase-, DNase-free water. To quantify mt tRNA levels, one-step qRT-PCRs were performed in an Applied Biosystems Step-One Real-Time PCR System. 5 μl of 1/10 diluted RNA samples were reverse-transcribed and amplified by qPCR in 12 μl of total volume reaction containing specific primers (Sigma; Supplementary Table S1), Power SYBR Green PCR Master Mix, MultiScribe Reverse Transcriptase, and RNase Inhibitor (all from Applied Biosystems), according to manufacturer’s instructions. Amplification efficiency values were very close to 100%. To quantify mt tRFs levels, 5 μl of 1/10 diluted RNA samples were reverse-transcribed and amplified by qPCR as described in section “RNA Isolation and qRT-PCR”. Relative quantitation of mt tRNAs and mt tRFs levels in mitochondria and cytoplasm fractions was calculated by the fold enrichment method 2^-[Ct(Protein)-Ct(IgG)].

### High-Resolution Respirometry in Intact Cells Using Oxygraph-2K (Oroboros)

Oxygen consumption rate (OCR) in oligonucleotides-transfected and non-transfected cybrid cells was measured using a high-resolution respirometer (Oxygraph-2k, Oroboros Instruments, Innsbruck, Austria). 80% confluent cells were detached at 37°C with trypsin–EDTA and resuspended in fresh growth media at a concentration of 2,000,000 cells/mL. Each cell type was analyzed in a 2 ml Oxygraph chamber. A real-time measurement of the oxygen consumption rate (OCR) was performed at 37C° in each chamber at basal conditions and after sequential addition of inhibitors for the different mitochondrial respiratory complexes: oligomycin (2.5 μg/ml) to inhibit complex V (to assess ATP-linked respiration and leak rate), carbonyl cyanide p-trifluoromethoxyphenylhydrazone (CCCP) uncoupler with stepwise titration in 2.5 to 1.5 μM increments (to assess maximal electron transport system respiratory capacity rate and reserve capacity), rotenone (0.5 μM) to inhibit complex I, and antimycin A (2.5 μM) to inhibit complex III (to assess non-mitochondrial respiratory capacity). Data were analyzed using DatLab7 (Oroboros, Austria) software.

### Statistical Analysis

Statistical analysis was performed using Student’s *t*-test and was conducted using GraphPad Prism 8 (GraphPad Software, Inc., San Diego, CA). The statistically significant differences between the means were indicated by asterisks (^*^p < 0.05, ^**^p < 0.01 or ^***^p < 0.001) and non-significant differences by n.s.

## Results

### The miRNA Pathway Components Dicer and Ago2 Are present in Mitochondria of WT and MELAS Cybrids

**Figure 1:**
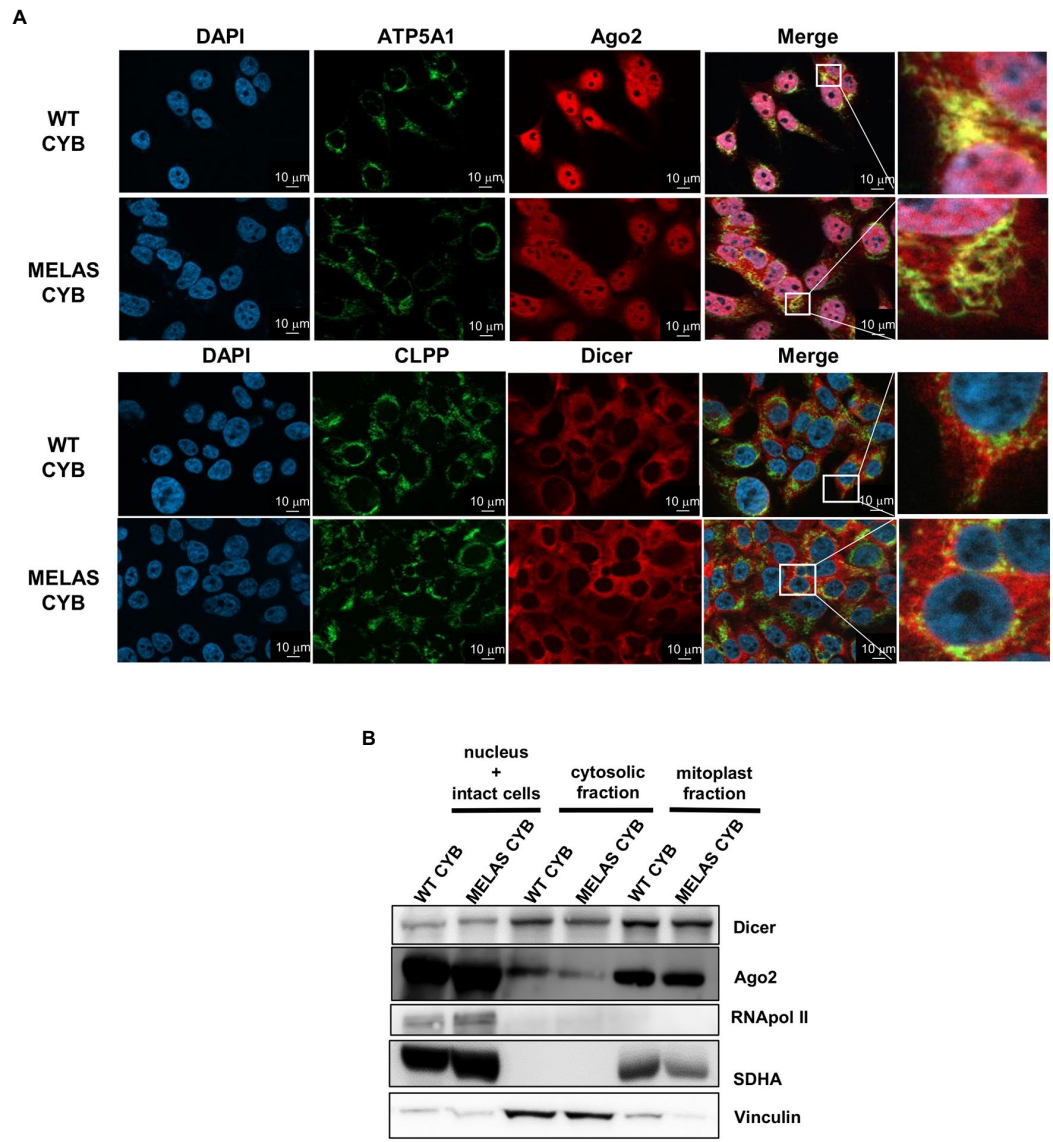
Partial localization of Dicer and Ago2 in the mitochondrial fraction of WT and MELAS cybrid cells. **(A)** Immunofluorescence microscopy analysis of endogenous Dicer and Ago2 subcellular localization in WT and MELAS cybrid cells. Nuclei were stained with DAPI (blue), and mitochondria were immunodetected with anti-ATP5A1 (OXPHOS Complex V subunit) or anti-CLPP (a serine protease located in the mitochondrial matrix). Scale bars, 10 μm. **(B)** Representative immunoblots of Dicer and Ago2 in subcellular fractions of WT and MELAS cybrid cells. Dicer and Ago2 levels were determined in nucleus and intact cells, cytosol and mitoplasts enriched fractions together with respective markers (Pol II (nucleus), Vinculin (cytoplasm), and SDHA (mitochondria)).

Given that we previously reported (i) a dependence of mt tRF levels on the components of miRNA pathway Dicer and Ago2 and (ii) a major accumulation of mt tRFs in mitochondria from osteosarcoma 143B (WT and MELAS) cybrids (Meseguer et al., 2019), we decided to investigate whether those proteins are located within mitochondria of these cells. We performed immunofluorescence staining in WT and MELAS cybrids using antibodies against Ago2 and Dicer, together with antibodies against the mitochondrial proteins ATP5A1 and CLPP, and we visualized the cells by Apotome microscopy. The confocal-like images showed a partial localization of Ago2 (Figure 1A, top panels) and Dicer (Figure 1A, below panels) to mitochondria in both WT and MELAS cybrids. To further investigate the location of Dicer and Ago2 within the cell, we also completed subcellular fractionation experiments. To evaluate an internal mitochondrial localization, a Digitonin treatment of the mitochondria-enriched fraction was included in the protocol, since this non-ionic detergent is able to solubilize proteins in the mitochondrial outer membrane (MOM), providing a mitoplast (mitochondrial inner membrane (MIM) + Matrix) enriched fraction. Purity of cytosolic and mitoplast fractions was monitored by immunodetection of the cytoplasm marker vinculin and the mitochondrial marker SDHA (Figure 1B). In these experiments, we detected both Dicer and Ago2 not only in the cytoplasm but also in mitoplasts of WT and MELAS cybrid cells.

Therefore, we confirmed by two experimental approaches the presence of both proteins inside the mitochondria in osteosarcoma 143B cybrid cells.

### Dicer and Ago2 May Interact With Mitochondrial tRNAs and tRFs

**Figure 2:**
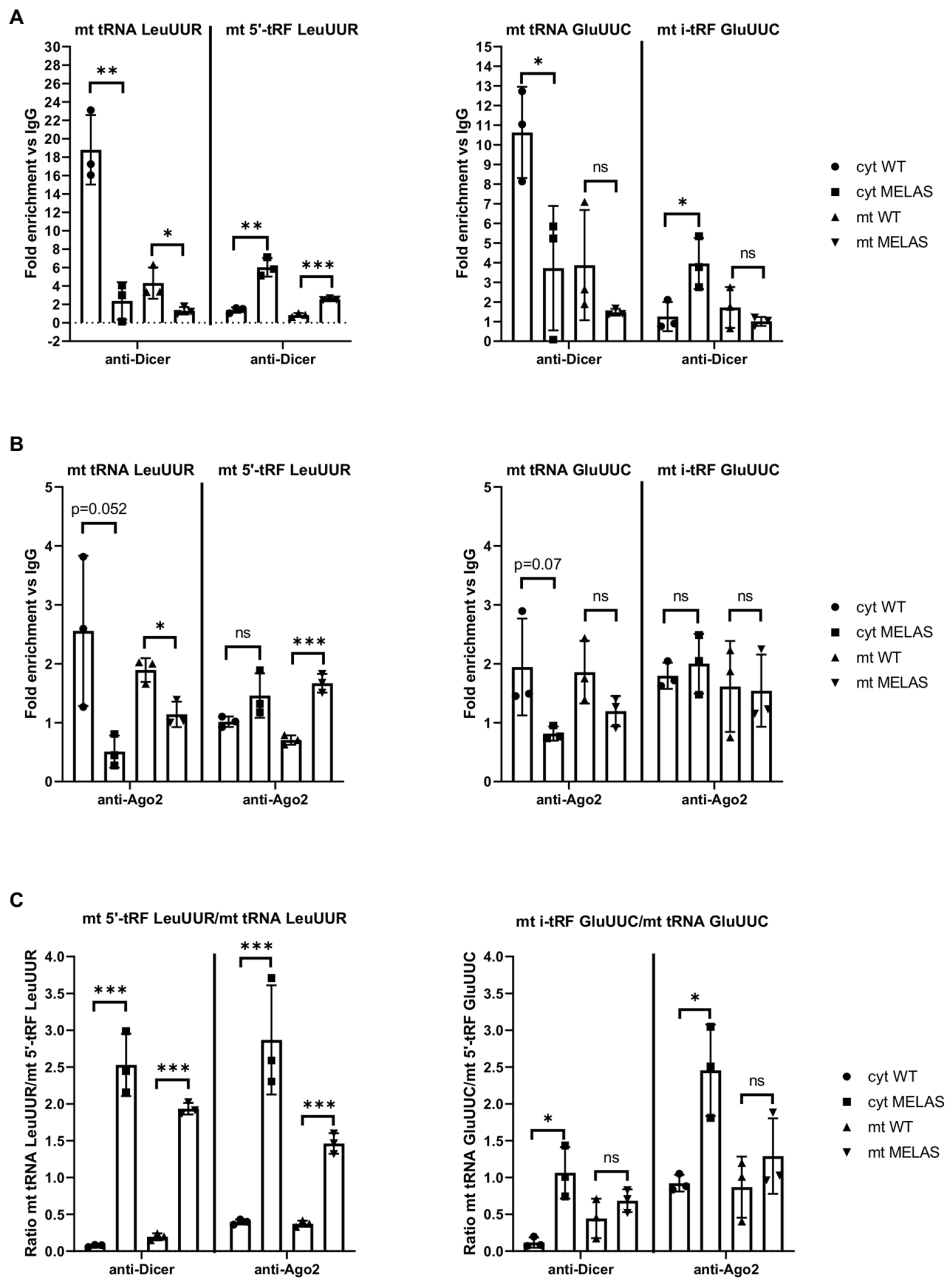
mt tRNAs may recruit Dicer and Ago2. (A and B) RT-qPCR analysis of the expression of mt tRNAs LeuUUR, and GluUUC and mt tRFs, mt 5′-tRF LeuUUR and mt i-tRF GluUUC in Dicer **(A)** and Ago2 **(B)** immunoprecipitates from cytosolic and mitochondrial fractions of WT and MELAS cybrids. Data are represented as fold change respect to values from IgG immunoprecipitates. **(C)** Ratio between fold enrichment values for a mt tRF and its parental mt tRNA in Dicer and Ago2 immunoprecipitates from cytosolic and mitochondrial fractions of WT and MELAS cybrids. Differences from control values were found to be statistically significant at ^*^p < 0.05, ^**^p < 0.01, ^***^p < 0.001.

Similar to other studies using different cell lines (Bandiera et al., 2011a,b; Das et al., 2012; Sripada et al., 2012; Tattikota et al., 2013; Zhang et al., 2014; Wang et al., 2015; Bose et al., 2020), we found that both components of the miRNA pathway, Dicer and Ago2, can be located in the mitochondria of osteosarcoma 143B cybrids. Furthermore, we had previously shown that silencing of Dicer and Ago2 in cybrids reduced the expression of the tested mt tRFs (Meseguer et al., 2019). The next step was to provide more evidence on whether these proteins participate in mt tRF biogenesis and in which cellular compartment/s this could take place. To this end, we immunoprecipitated Dicer and Ago2 from the cytosolic and mitochondrial fractions of WT and MELAS cybrids and analyzed whether selected mt tRNAs (mt tRNA LeuUUR and GluUUC) and their derivative mt tRFs species (mt 5′-tRF LeuUUR and mt i-GluUUC) were bound to these proteins. RT-qPCR analysis of cytosolic fractions showed lower levels of mt tRNA LeuUUR bound to Dicer in MELAS than in WT cybrids (Figure 2A, left panel). Although mt tRNAs LeuUUR bound to Dicer were less detectable in mitochondrial fractions, we also observed a decrease in MELAS versus WT cells. Interestingly, the opposite result was observed when mt 5′-tRFs LeuUUR were evaluated in both cellular fractions. With respect to the levels of mt tRNA GluUUC and its derivative mt tRF specie (mt i-GluUUC), they showed an equivalent pattern to mt tRNA LeuUUR and mt 5′-tRF LeuUUR in cytosolic fractions but in mitochondria there were no significant differences (Figure 2A, right panel). On the other hand, Ago2 immunoprecipitations were less effective since the fold enrichment respect to IgG was low in almost all cases (Figure 2B). Even so, a pattern similar to that of Dicer immunoprecipitates was observed for mt tRNA LeuUUR and mt 5′-tRF LeuUUR (Figure 2B, left panel). All together, we found a general increase of the mt tRF/mt tRNA ratio in the Dicer and Ago2 immunoprecipitates from both fractions in favor of MELAS (Figure 2C). The different mt tRF/mt tRNA ratio between WT and MELAS cells indicate a differential recruitment of mt tRNAs versus mt tRFs by Dicer and Ago2 and could be the result of the stimulation of the biogenesis process of the mt tRFs in the MELAS condition: (i) the increase of Dicer-dependent digestion of mt tRNAs in MELAS cells would reduce their presence in Dicer’s immunoprecipitates from these cells while increasing the presence of their mt tRF derivatives, (ii) if Ago2 were facilitating the transfer of mt tRNAs to Dicer and/or the reception of mt tRF derivatives from digestion, a stimulated biogenesis would also shift the mt tRNA-mt tRF equilibrium in favor of mt tRFs. Thus it would explain the increase of mt tRFs in Ago2 immunoprecipitates in MELAS compared to WT. However, other experimental approaches including knock out and knock in cybrids for Dicer and Ago2 are needed to validate this hypothesis.

### The Treatment With a 5′-tRF LeuUUR Mimic Partially Restores Mitochondrial Respiration in MELAS Cybrids

**Figure 3:**
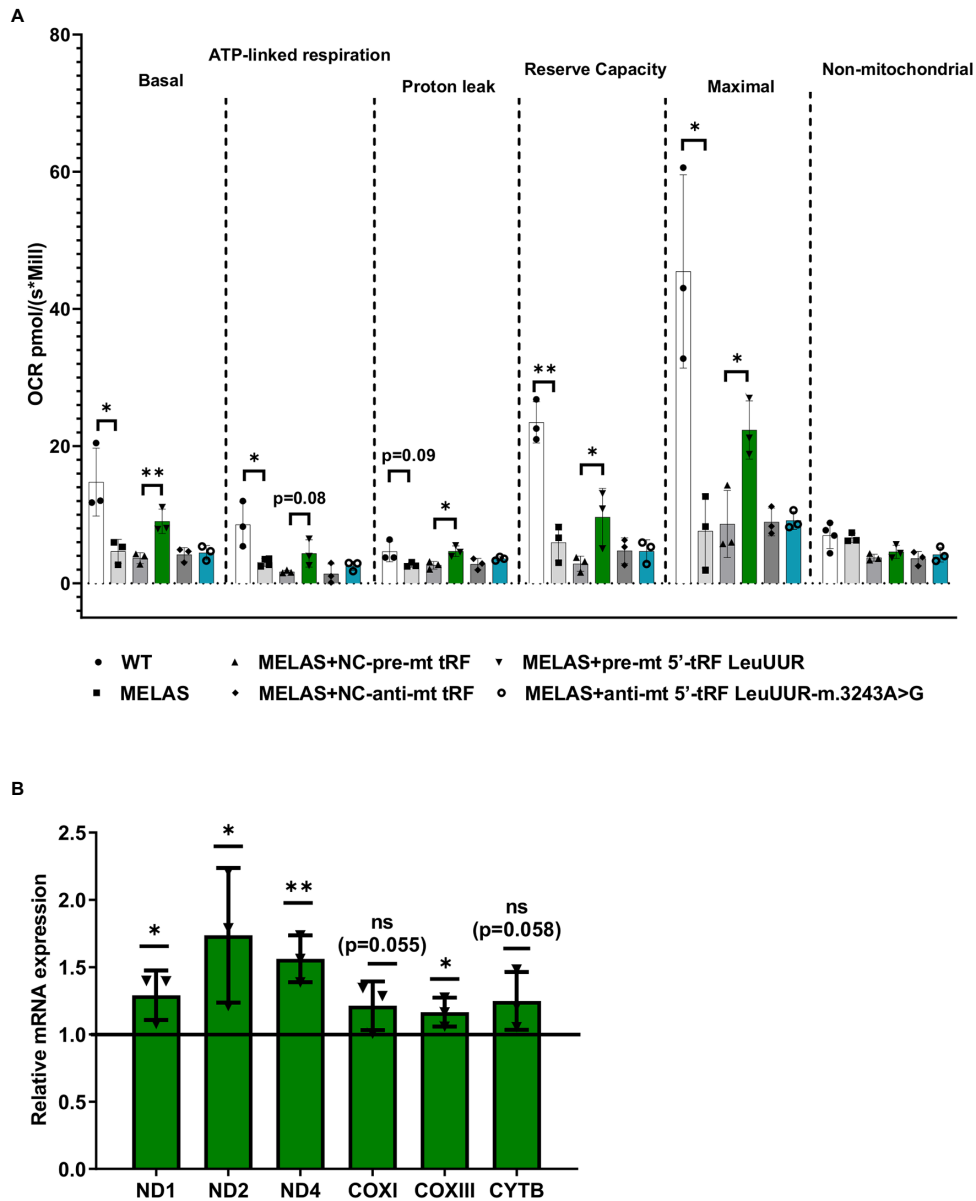
Over-expression of mt tRNA LeuUUR fragment (mt 5′-tRF LeuUUR) increases mitochondrial respiration in MELAS cybrids. **(A)** Analysis of oxygen consumption rate (OCR) of WT and MELAS cybrids, and MELAS cybrids transfected with an oligonucleotide mimic of mt-tRNA LeuUUR fragment (mt 5′-tRF LeuUUR), an oligonucleotide antisense against mt-tRNA LeuUUR-m.3243A>G fragment (mt 5′-tRF LeuUUR-m.3243A>G) and their respective controls. OCR was measured under basal conditions and after sequential addition of different OXPHOS inhibitors: oligomycin, carbonyl cyanide p-trifluoromethoxyphenylhydrazone (CCCP), rotenone, and antimycin A. The scatter plot shows basal OCR (determined as the difference between OCR before oligomycin and OCR after rotenone/antimycin incorporation A), ATP-linked OCR (difference between OCR before and after oligomycin), proton leak (difference between basal OCR and ATP-linked OCR), reserve capacity (difference between the CCCP-stimulated rate and basal OCR), non-mitochondrial OCR (OCR after rotenone and antimycin A treatment), and maximal OCR (difference between OCR after CCCP and non-mitochondrial OCR). **(B)** RT-qPCR analysis of the expression of *ND1*, *ND2*, *ND4, COXI, COXIII*, and *CYTB* mRNAs in MELAS cybrids transfected with an oligonucleotide mimic of mt-tRNA LeuUUR fragment (mt 5′-tRF LeuUUR) with respect to negative control-transfected cells. Data are represented as fold change respect to values from control samples. The horizontal bar represents the media value for control samples. Differences from control values were found to be statistically significant at ^*^p < 0.05 and ^**^p < 0.01.

mt tRFs may be biologically relevant as we have previously demonstrated that one of them (mt i-tRF GluUUC) downregulates the expression of its target (MPC1) and, accordingly, has an effect on a phenotypic trait of the disease (the accumulation of extracellular lactate; Meseguer et al., 2019). In that work we also found that two significantly altered mt tRFs are derived from the mt tRNA affected by the MELAS mutation (m.3243A>G), mt 5′-tRF LeuUUR, and mt 5′-tRF LeuUUR-m.3243A>G. Here, we decided to evaluate the effect of modulating the levels of those mt tRFs, by treatment with specific oligonucleotides, on another important phenotypic characteristic of MELAS cells, a defective mitochondrial respiration. To this end, we transfected MELAS cybrids with one of the following: a mimic oligonucleotide of the wild-type mt tRNA LeuUUR fragment (pre-mt 5′-tRF LeuUUR), an antisense oligonucleotide of mutant mt tRNA LeuUUR fragment (anti-mt 5′-tRF LeuUUR-m.3243A>G) or their respective negative controls. The oxygen consumption rate (OCR) was monitored in intact cells by the High-resolution respirometer Oroboros instrument, in basal conditions and using a combination of inhibitors to analyze different respiratory states. We found that basal, ATP-linked, proton leak, reserve capacity, and maximal OCR were significantly increased in MELAS cells transfected with the mt-tRF LeuUUR mimic with respect to negative control-transfected cells (Figure 3A). However, we did not observe any effect after transfecting the cells with the anti-mt 5′-tRF LeuUUR-m.3243A>G as compared with its negative control. These results demonstrate that the mt 5′-tRF LeuUUR has also a biological relevance since its levels affect the mitochondrial respiration.

According to literature, tRFs can act as post-transcriptional regulators of specific mRNAs, as miRNAs do (Yeung et al., 2009; Kuscu et al., 2018). It has also been reported that sncRNAs encoded by mtDNA may affect mitochondrial gene expression (Ro et al., 2013). Using RNAhybrid, a prediction tool of microRNA/target RNA duplexes, we studied the putative binding sites to mtDNA-encoded genes for the mt 5′-tRF LeuUUR. We found at least 100 interactions along mtDNA (Supplementary Figure S1A), almost 50% of which were located in mt mRNAs codifying for OXPHOS subunits (Supplementary Figure S1B). We next examined the levels of several mt mRNAs in MELAS cells transfected with the mt 5′-tRF LeuUUR mimic and compared them to those of MELAS cells transfected with the control mimic. We found that some mitochondrial transcripts, mainly those encoding for Complex I subunits, increased significantly (Figure 3B) when the levels of the wild-type fragment were enhanced (Supplementary Figure S2). These results support the idea that mt 5′-tRF LeuUUR participates in the regulation of the expression of mt DNA-encoded genes. However, we cannot exclude an indirect regulation by a nuclear-encoded target of mt 5′-tRF LeuUUR.

## mt tRFs Are Detected in Different MELAS Patient Samples

**Figure 4:**
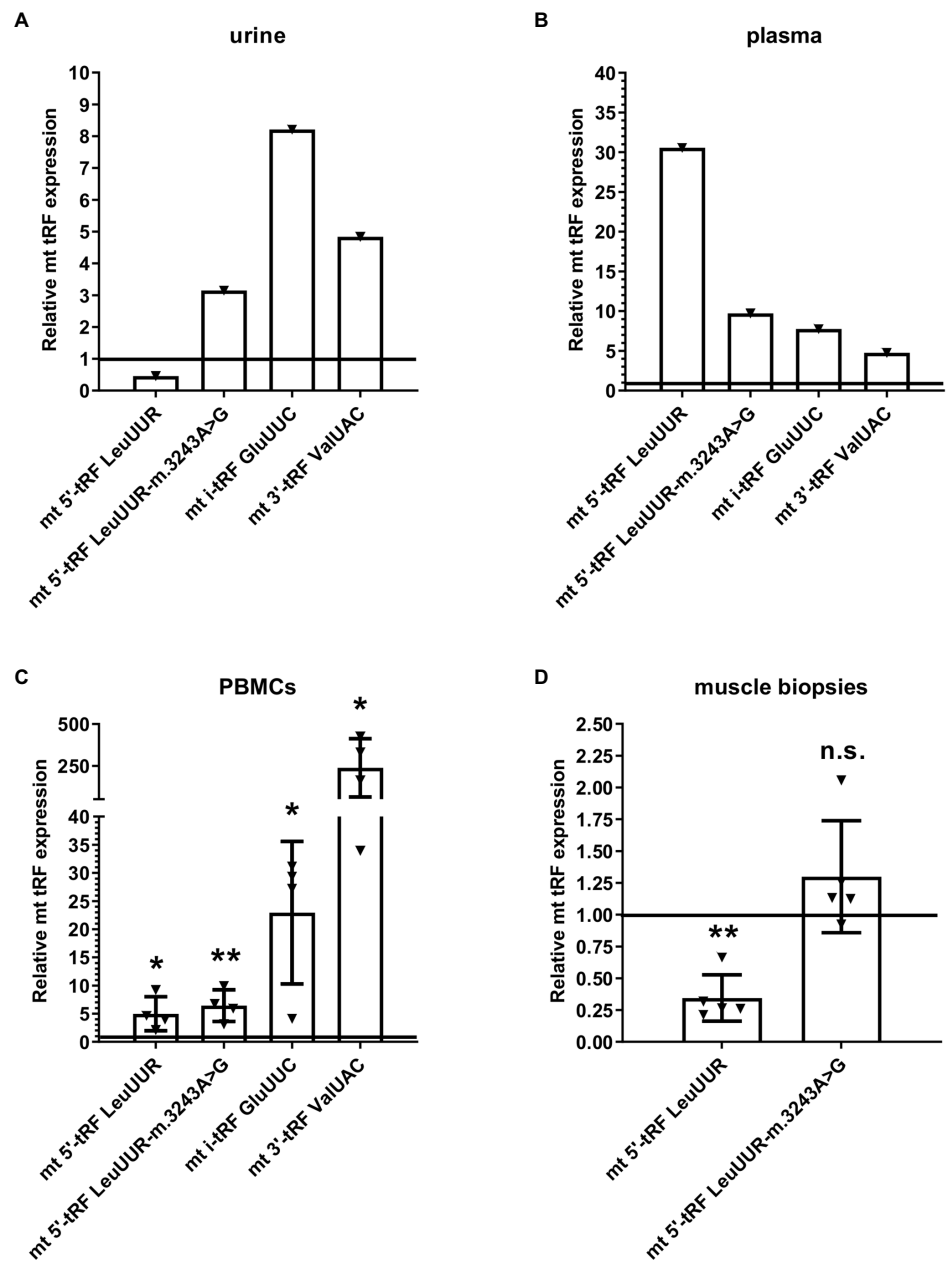
Expression profile of mt 5′-tRF LeuUUR, mt 5′-tRF LeuUUR-m.3243A>G, mt i-tRF GluUUC, and mt 3′-tRF ValUAC in a small cohort of diverse samples from MELAS patients and controls. RT-qPCR analysis of the expression of mt 5′-tRF LeuUUR, mt 5′-tRF LeuUUR-m.3243A>G, mt i-tRF GluUUC and mt 3′-tRF ValUAC in biofluids (urine and plasma), PBMCs, and muscle tissues from MELAS patients as compared to controls. Data are represented as fold change respect to values from control samples. Each triangle represents an individual and the horizontal bar the media value for control group. Differences from control values were found to be statistically significant at ^*^p < 0.05 and ^**^p < 0.01.

Considering that the levels of mt i-tRF GluUUC, mt 5′-tRF LeuUUR-m.3243A>G, and mt 3′-tRF ValUAC are increased in MELAS cybrids and fibroblasts compared to controls and those of mt 5′- tRF LeuUUR (the wild-type version of the fragment) are reduced (Meseguer et al., 2019), we wanted to study whether it is possible to detect this tRF signature in different types of MELAS samples. We evaluated the levels of those mt tRFs in a small but diverse cohort of MELAS and control samples, specifically, biofluids, such as urine and plasma from one MELAS and two control individuals, PBMCs from four MELAS and two control individuals, and muscle tissue from five MELAS and two control individuals. RT-qPCR analysis showed that in MELAS the expression of mt 5′-tRF LeuUUR-m.3243A>G, mt i-tRF GluUUC, and mt 3′-tRF ValUAC was increased in urine, plasma, and PBMCs (Figure 4). Interestingly, mt 5′- tRF LeuUUR levels were reduced in urine (Figure 4A) similarly to what was found in MELAS cybrids and fibroblasts (Meseguer et al., 2019) but were increased in plasma and PBMCs (Figures 4B,C). Due to the limited amount of RNA isolated from muscle tissue samples, we were only able to analyze mt 5′-tRF LeuUUR-m.3243A>G and mt 5′-tRF LeuUUR. We found low levels of mt 5′-tRF LeuUUR in muscles, and only one sample showed high levels of mt 5′-tRF LeuUUR-m.3243A>G (Figure 4D).

These results, despite the limited number of individuals in the study, hint that the tRF signature found in cybrids and fibroblasts can also be present in other MELAS samples, including biofluids, suggesting that these elements are good non-invasive biomarker candidates to be explored in depth in a study with a larger cohort. Furthermore, blood samples (plasma and PBMCs) showed increased levels of mt 5′-tRF LeuUUR in MELAS patients, a fact that could be interesting to analyze in more detail.

## Discussion

In this study, we demonstrated that the components of miRNA pathway Dicer and Ago2 are located in a fraction of mitochondria from WT and MELAS cybrids. Previously, other groups had also found Dicer and Ago2 in these organelles from other cell lines (Bandiera et al., 2011a,b; Das et al., 2012; Sripada et al., 2012; Tattikota et al., 2013; Zhang et al., 2014; Wang et al., 2015; Bose et al., 2020). However, this finding has not always been confirmed (Das et al., 2012; Ro et al., 2013), probably due to differences in the cell type and/or the preparation of the sample used in those other studies. The immunoprecipitation of Dicer in cytosolic and mitochondrial fractions provided mt tRNA and tRFs among the RNA pool bound to Dicer. In these *in vitro* experiments, tested mt tRNAs were more abundant in Dicer immunoprecipitates from WT fractions than from MELAS, while the opposite occurred for their derived tRFs. Although further experiments are needed to confirm whether (i) this is a direct interaction and (ii) this occurs *in vivo*, it is tentative to speculate that Dicer participates in the biogenesis of mt tRFs and that its activity is specially stimulated in the MELAS condition in basis of the increased ratio mt tRF/mt tRNA compared to WT. The immunoprecipitation of Ago2 did not provide results as clear as Dicer’s but a similar tendency was observed at least for mt tRNA LeuUUR and its derived mt tRF. In the previous work, we postulated two putative mechanisms for mt tRF biogenesis which depended on Dicer and Ago2 localization within the cell. In case there were only cytosolic Dicer and Ago2 proteins, we suggested that mt tRNA molecules would be exported out of the mitochondria and processed by Dicer in the cytoplasm to generate mt tRFs. A cytosolic fraction of mt tRFs would be loaded onto Ago2 for the silencing of nuclear-encoded genes, like MPC1. In a mechanism that considers a mitochondrial fraction of Dicer and Ago2, we proposed that mt tRNAs could be processed by Dicer within the mitochondria to generate mt tRFs that would be loaded onto mt Ago2 proteins. These RNA-Ago2 complexes could participate in the regulation of the expression of mt DNA-encoded genes and/or a fraction of the mitochondrially processed mt tRFs could be transported to the cytoplasm for the regulation of nuclear-encoded genes. Our results from Dicer immunoprecipitations suggest that this enzyme operates both in the cytoplasm and inside the mitochondria, providing mt tRFs that could be participating in the post-transcriptional regulation of nDNA- and mtDNA-encoded gene expression, respectively (Supplementary Figure S3). The idea of a mt tRF biogenesis mechanism that functions according to the target origin is supported by the fact that mt i-tRF GluUUC, which regulates the expression of a nuclear-encoded gene (MPC1), was only detectable in the cytosolic fraction of MELAS in detrimental of its parental mt tRNA in the same fraction. Further experiments are needed to demonstrate whether the proposed biogenesis mechanism is correct.

We also explored the therapeutic strategy of administering a mt 5′-tRF LeuUUR mimic and mt 5′-tRF LeuUUR-m.3243A>G antagonist oligonucleotides to MELAS cells. We found that treatment with the mt 5′-tRF LeuUUR mimic improved the mitochondrial respiration, a phenotypic trait of the disease, and that it was accompanied by an increase of the expression of some mitochondrial transcripts, mainly those encoding for Complex I subunits. These results support the idea that mt 5′-tRF LeuUUR participates in the regulation of the expression of mt DNA-encoded genes and, consequently, has an effect on mitochondrial respiration. Perhaps, it would be interesting to analyze whether the mitochondrial respiration improvement increases when the mitochondrial internalization of the mt 5′-tRF LeuUUR is enhanced, using a mitochondrial deliver system like mitoPORTER (Yamada et al., 2008, 2020).

Finally, we explored the potential of mt tRFs as non-invasive biomarkers of MELAS disease. In particular, we analyzed whether the tRF signature observed in MELAS cybrids and fibroblasts [increased levels of mt i-tRF GluUUC, mt 5′-tRF LeuUUR-m.3243A>G, and mt 3′-tRF ValUAC and reduced levels of mt 5′- tRF LeuUUR (the wild-type version of the fragment) compared to controls] was also exhibited by a small group of diverse samples from MELAS patients including biofluids like urine and plasma. The urine sample showed the same tRF signature as MELAS cybrids and fibroblasts while blood samples (PBMCs and plasma) differed only in mt 5′- tRF LeuUUR levels, which were increased in these MELAS specimens. Based on these results, it would be interesting to explore in a study with a larger cohort of MELAS samples whether mt tRFs detection in biofluids could be an effective non-invasive diagnostic system.

In summary, this short study provides preliminary data on the biogenesis mechanism of mt tRFs and highlights the necessity to keep exploring the biological relevance and the therapeutic and diagnostic potential of mt tRFs.

## Data Availability Statement

The raw data supporting the conclusions of this article will be made available by the authors, without undue reservation.

## Ethics Statement

The studies involving human participants were reviewed and approved by Ethics Committee of Foundation for the Promotion of Health and Biomedical Research of Valencia Region, FISABIO (Valencia, Spain) and Committee of Milano Area 2, Fondazione IRCCS Ca′ Granda Ospedale Maggiore Policlinico (Milan, Italy). The patients/participants provided their written informed consent to participate in this study.

## Author Contributions

SM designed the study and wrote the paper. SM and M-PR performed the experiments. All authors reviewed the manuscript. All authors contributed to the article and approved the submitted version.

## Funding

This work has been supported by grant GV/2020/191 from the Valencian Ministry of Innovation, Universities, Science and Digital Society to Salvador Meseguer.

## Conflict of Interest

The authors declare that the research was conducted in the absence of any commercial or financial relationships that could be construed as a potential conflict of interest.

## Publisher’s Note

All claims expressed in this article are solely those of the authors and do not necessarily represent those of their affiliated organizations, or those of the publisher, the editors and the reviewers. Any product that may be evaluated in this article, or claim that may be made by its manufacturer, is not guaranteed or endorsed by the publisher.

## Supplementary Material

Supplementary Table S1 Oligonucleotides used in this work.

Supplementary Figure S1 Potential binding sites of mt 5′-tRF LeuUUR to the mitochondrial DNA-encoded transcripts. **(A)** Binding sites for the mt 5′-tRF LeuUUR along the mitochondrial DNA and the minimum free energy of these hybridizations provided by RNAhybrid using a threshold of -15Kcal/mol. **(B)** Number of binding sites per mitochondrial transcript encoding for an OXPHOS subunit.

Supplementary Figure S2 Levels of mt 5′-tRF LeuUUR in MELAS cells transfected with the mt-tRF LeuUUR mimic. RT-qPCR analysis of the expression of mt 5′-tRF LeuUUR in MELAS cells transfected with the mt-tRF LeuUUR mimic with respect to negative control-transfected cells. Data are represented as fold change respect to values from control samples. Differences from control values were found to be statistically significant at ^*^*p* < 0.001.

Supplementary Figure S3 Proposed model of mt tRF biogenesis. (I) mt tRNA molecules would be exported out of the mitochondria and processed by Dicer in the cytoplasm to generate mt tRFs. Mitochondrial and cytoplasmic fractions of Ago2 proteins could be involved in the transport of mt tRNAs from mitochondria to cytosolic Dicer and mt tRFs generated in the cytoplasm would be loaded onto cytosolic Ago2 for the silencing of nuclear-encoded genes (OXPHOS subunits and/or other mitochondrial-related proteins). (II) mt tRNAs could be processed by Dicer within the mitochondria to generate mt tRFs that would be loaded onto mt Ago2 proteins. These mt tRF-Ago2 complexes could participate in the regulation of the expression of mt DNA-encoded genes (OXPHOS subunits).
