# Supplementary figures and images for: mt tRFs, New Players in MELAS Disease
Source: Front Physiol. 2022 Feb 22;13:800171. doi: 10.3389/fphys.2022.800171 (PMC8902416; doi:10.3389/fphys.2022.800171)

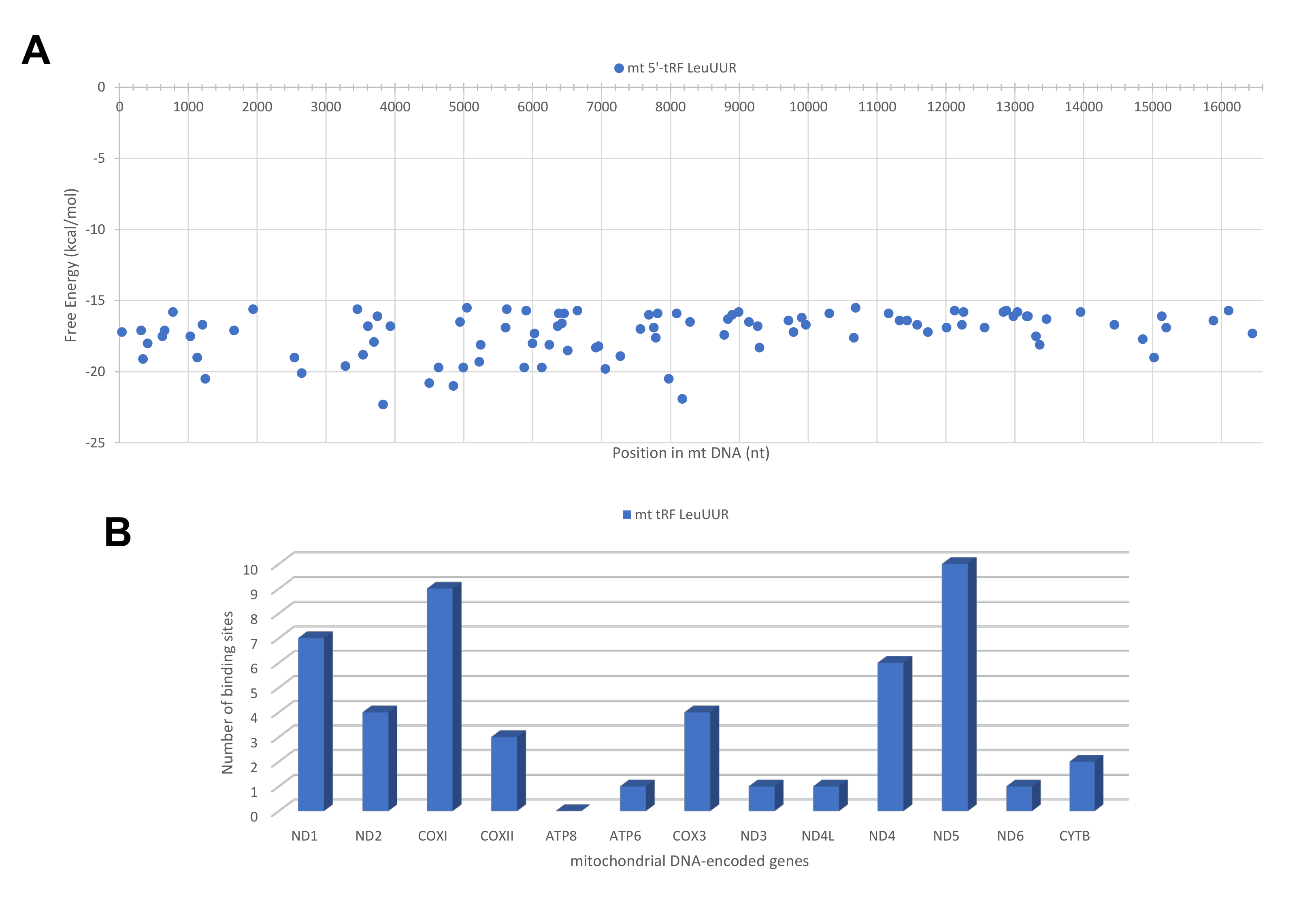

Supplement: Supplementary Figure S1 — Potential binding sites of mt 5′-tRF LeuUUR to the mitochondrial DNA-encoded transcripts. (A) Binding sites for the mt 5′-tRF LeuUUR along the mitochondrial DNA and the minimum free energy of these hybridizations provided by RNAhybrid using a threshold of -15Kcal/mol. (B) Number of binding sites per mitochondrial transcript encoding for an OXPHOS subunit. [file Image_1.TIF]

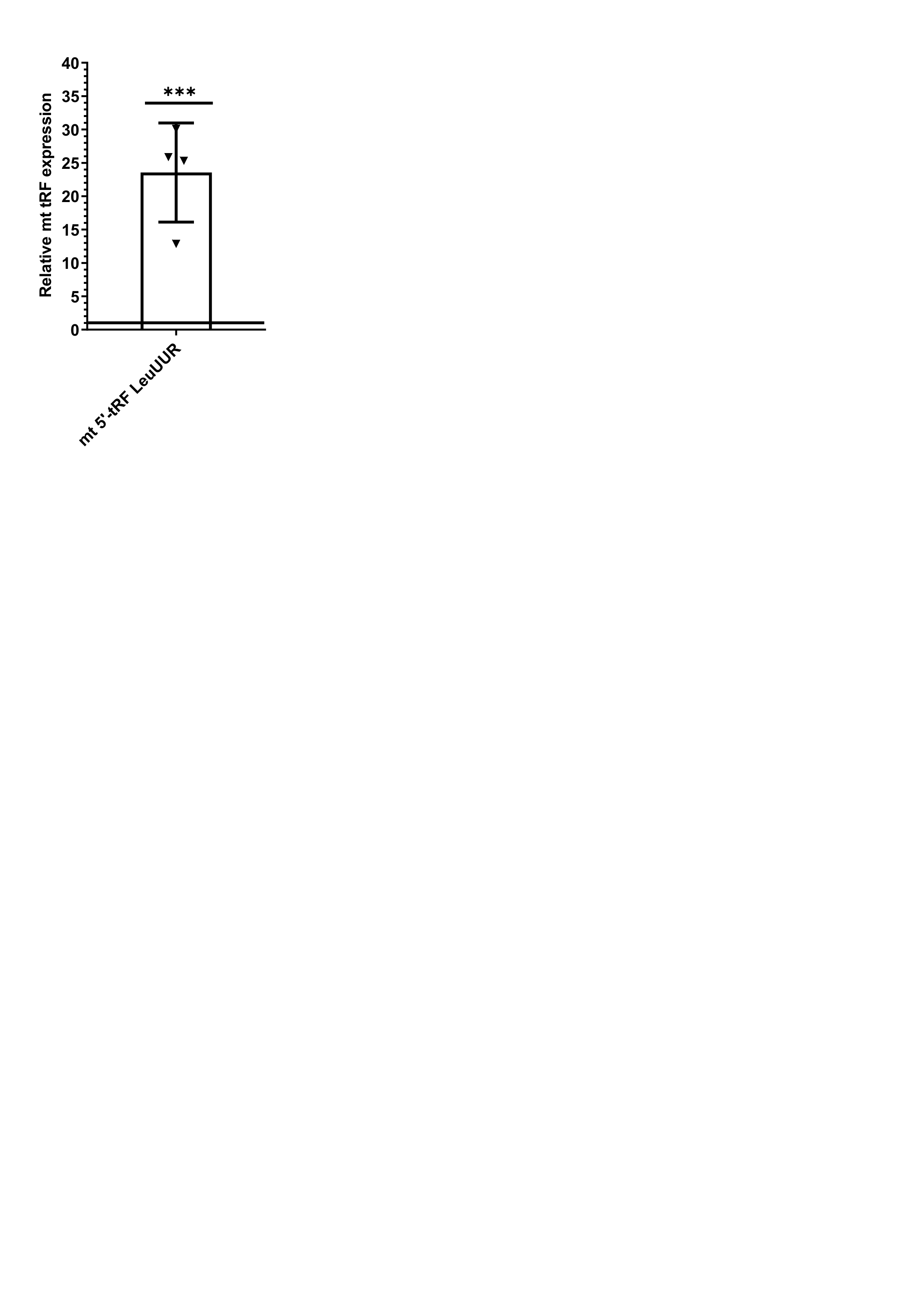

Supplement: Supplementary Figure S2 — Levels of mt 5′-tRF LeuUUR in MELAS cells transfected with the mt-tRF LeuUUR mimic. RT-qPCR analysis of the expression of mt 5′-tRF LeuUUR in MELAS cells transfected with the mt-tRF LeuUUR mimic with respect to negative control-transfected cells. Data are represented as fold change respect to values from control samples. Differences from control values were found to be statistically significant at *p < 0.001. [file Image_2.TIF]

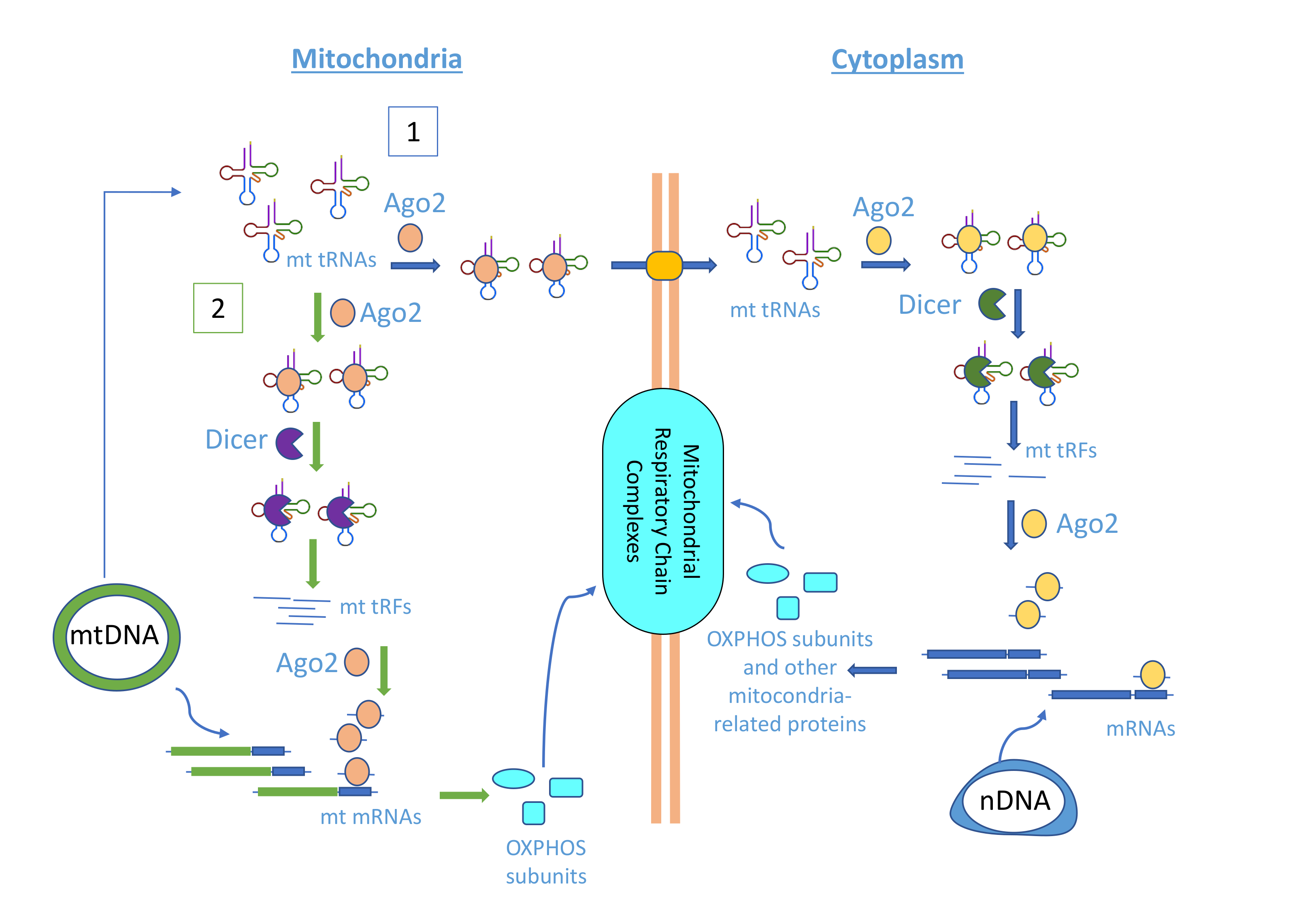

Supplement: Supplementary Figure S3 — Proposed model of mt tRF biogenesis. (I) mt tRNA molecules would be exported out of the mitochondria and processed by Dicer in the cytoplasm to generate mt tRFs. Mitochondrial and cytoplasmic fractions of Ago2 proteins could be involved in the transport of mt tRNAs from mitochondria to cytosolic Dicer and mt tRFs generated in the cytoplasm would be loaded onto cytosolic Ago2 for the silencing of nuclear-encoded genes (OXPHOS subunits and/or other mitochondrial-related proteins). (II) mt tRNAs could be processed by Dicer within the mitochondria to generate mt tRFs that would be loaded onto mt Ago2 proteins. These mt tRF-Ago2 complexes could participate in the regulation of the expression of mt DNA-encoded genes (OXPHOS subunits). [file Image_3.TIF]
